# VSFlow: an open-source ligand-based virtual screening tool

**DOI:** 10.1186/s13321-023-00703-1

**Published:** 2023-03-31

**Authors:** Sascha Jung, Helge Vatheuer, Paul Czodrowski

**Affiliations:** 1 Department of Chemistry and Chemical Biology, TU Dortmund University, Otto-Hahn-Straße 6, 44227 Dortmund, Germany; 2 Department of Chemistry, Johannes Gutenberg University Mainz, Duesbergweg 10-14, 55128 Mainz, Germany

**Keywords:** Virtual screening, Substructure, Fingerprints, Shape, Python, RDKit

## Abstract

Ligand-based virtual screening is a widespread method in modern drug design. It allows for a rapid screening of large compound databases in order to identify similar structures. Here we report an open-source command line tool which includes a substructure-, fingerprint- and shape-based virtual screening. Most of the implemented features fully rely on the RDKit cheminformatics framework. VSFlow accepts a wide range of input file formats and is highly customizable. Additionally, a quick visualization of the screening results as pdf and/or pymol file is supported.

**Graphical Abstract:**

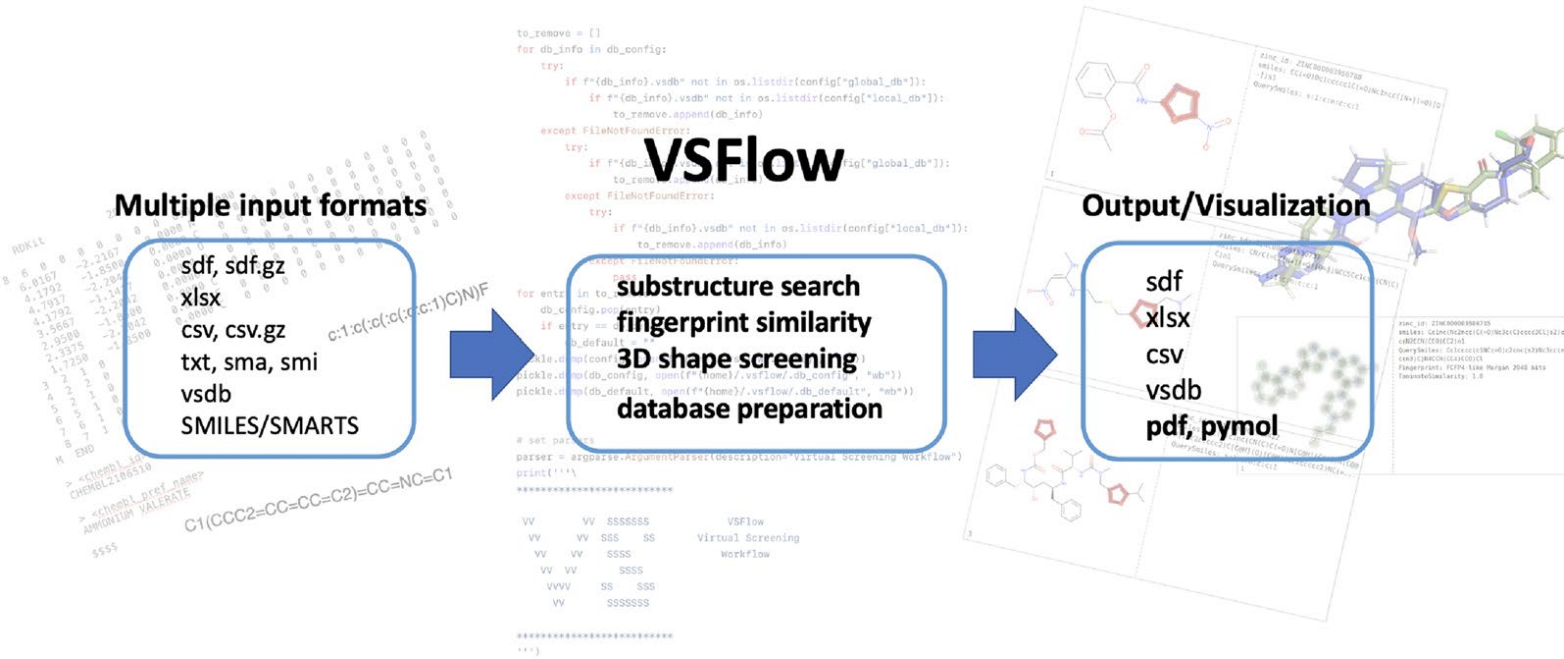

## Introduction

Virtual screening approaches are extensively used computational methods in modern drug discovery projects and they often replace or help to reduce more expensive and time-consuming high-throughput screenings nowadays [1]. There are two major categories of screening approaches: ligand-based and structure-based methods [2].

Ligand-based methods are typically used if no X-ray structure of the target receptor is available. A single compound or a set of compounds known to bind to a specific target or to be active in a functional assay is typically used as the template to identify similar compounds in a large virtual database. In general, similarity can be evaluated on the basis of 2D and 3D molecular representations [3]. The classical 2D chemical similarity representations is based on molecular fingerprints (e.g. circular fingerprints, topological fingerprints, substructure fingerprints) transforming the molecular representation into a bit vector. The similarity between two vectors is then calculated with various similarity measures, most common is the Tanimoto coefficient. 3D similarity methods mainly consider the shape comparison of two molecules, typically extended by 3D pharmacophoric features, e.g. ROCS is considered the industry-leading commercial program for shape-based screenings [4].

Structure-based approaches, in most cases classical docking methods, are typically preferred if the target 3D structure information is available [5]. However, 2D ligand-based methods often require only a fraction of second for a single structure comparison task which allows to perform large screenings within a few hours even on a single, standard CPU. In contrast, docking methods are already considerably more resource demanding and time-consuming, not to mention more elaborated methods such as molecular dynamics simulations [6]. As a consequence, ligand-based methods are very attractive options for initial attempts to identify or filter relevant compounds in large and ultra-large virtual databases [7]. Furthermore, they are valuable tools to identify close analogues of known active compounds in a time efficient manner. In the last couple of years, several methods have been developed to screen non-enumerated chemical spaces up to 10^15^ compounds and beyond in seconds to minutes on standard hardware [8]. The most elaborated technique for large space screening are chemical fragment spaces with corresponding connection rules, e.g. BioSolveIT’s fragment spaces in connection with FTrees similarity implemented in their infiniSee software allows the screening of huge chemical spaces (e.g. Enamine REAL space) in seconds on standard hardware [9, 10].

There are many open-source web servers available for the screening of enumerated compound libraries using a variety of different structure- and ligand-based methods, recently reviewed by Singh et al. [11]. For example, many well-known databases such as ChEMBL, PubChem or ZINC include ligand-based similarity search functionalities with molecular fingerprints and/or substructure searches [12–14]. The web tool SwissSimilarity allows for the 2D fingerprint and 3D shape screening of common public databases and compound libraries of most commercial vendors such as Enamine or ChemDiv [15, 16]. Pharmit additionally offers the possibility to screen large databases based on pharmacophore queries [17].

Several standalone tools focusing on enumerated 2D ligand-based screening approaches are available, most of which are commercial products [8]. Prominent examples are Schrödinger‘s GPUSimilarity integrated in their LiveDesign suite using a GPU-powered server in the background, Arthor‘s NextMove software with a SMARTS-based pattern matcher and Andrew Dalke‘s chemfp command line tool [18–20].

To the best of our knowledge, there is no open-source command line tool available which is similar to the SwissSimilarity or Pharmit web server and which allows for the comprehensive screening of databases and library files using different 2D and 3D ligand-based screening approaches, all combined in one tool.

**Fig. 1:**
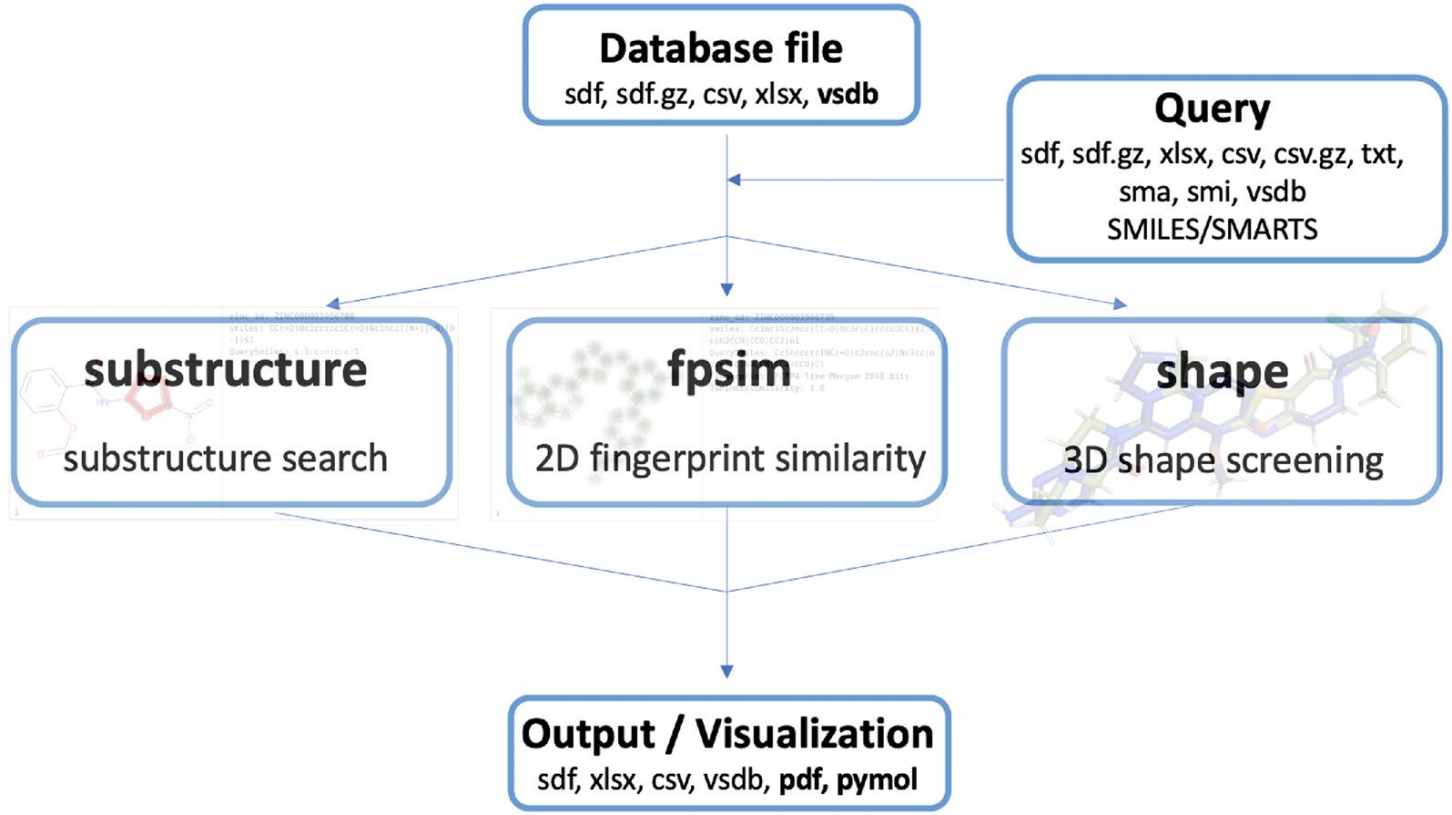
Different screening functionalities of VSFlow

In the following, we report an open-source command-line tool called “Virtual Screening WorkFlow” (VSFlow) written in Python and containing three different ligand-based screening modes. It relies on the open-source cheminformatics sofware RDKit [21]. VSFlow includes a substructure-based and fingerprint-based screening mode (2D) as well as a 3D shape-based screening mode (Fig. 1). Additionally, it possesses two tools for preparing and managing compound databases for virtual screening.

## Implementation

VSFlow is written in Python, is open-source and can be downloaded from https://github.com/czodrowskilab/VSFlow. It is licensed under the MIT license. As a prerequisite, a working installation of Anaconda or Miniconda is needed [22]. VSFlow including all dependencies can then be installed with the provided yml file as follows: 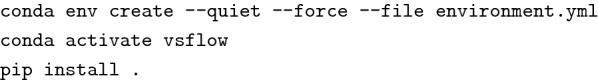 The Python dependencies are rdkit, xlrd, xlsxwriter, pdfrw, fpdf, pymol-open-source, molvs and matplotlib [23, 24]. VSFlow requires Python version 3.7 or higher.

VSFlow includes 5 separate tools: preparedb, substructure, fpsim, shape and managedb (Fig. 1). All functionalities of VSFlow can also be run in parallel on multiple cores/threads. Parallelization is implemented via Python’s built-in multiprocessing module.

### preparedb: prepare databases

**Fig. 2:**
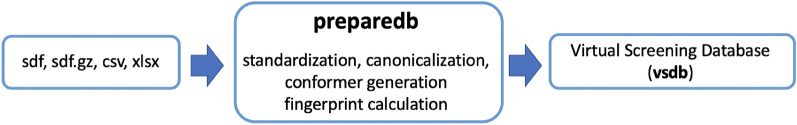
Preparedb functionality of VSFlow: prepare compound libraries for virtual screening

VSFlow contains a tool to prepare compound libraries for virtual screening (preparedb). It allows for standardization of the molecules, generation of fingerprints and generation of multiple conformers (Fig. 2). The output file is a “virtual screening database” (.vsdb) file. The vsdb file is a Python pickle file containing all information in a special Python dictionary format which significantly enhances loading speed compared to SD files, particularly relevant for larger databases. Standardization is done on the basis of the MolVS rules and includes charge neutralization, salt removal and optionally tautomer canonicalization [23]. Fingerprints are generated with the RDKit chemistry framework. Conformers are generated with the RDKit ETKDGv3 method and optimized with the MMFF94 forcefield [25]. The following options are available:

- standardize: standardizes molecules, removes salts and associated charges
- conformers: generates multiple 3D conformers for database molecules
- canonicalize: adds the canonical tautomer to the database
- fingerprint: generates the respective fingerprint for each molecule and stores it in the database

It is also possible to directly download the PDB ligands and the chembl database and store them as vsdb databases, e.g. 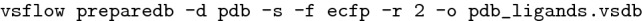


The above command will download all pdb ligands, standardize the molecules (-s argument), calculate the ECFP2 fingerprint (-f and -r argument) for every molecule and store it along with the molecule in the database (-o argument). You can repeat this for the ChEMBL database, e.g. with a different fingerprint: 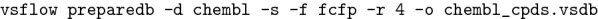

### substructure: substructure search

The substructure search (substructure) is performed based on the GetSubstructMatches() functionality available for RDKit Mol objects.

### fpsim: fingerprint similarity search

The fingerprint generation relies on the RDKit framework. All fingerprints currently implemented in the RDKit (Morgan, RDKit, Topological Torsion and Atom Pairs fingerprint and MACCS keys) are supported and different similarity measures (Tanimoto, Tversky, Cosine, Dice, Sokal, Russel, Kulczynski and McConnaughey similarity) can be used.

### shape: shape-based screening

**Fig. 3:**
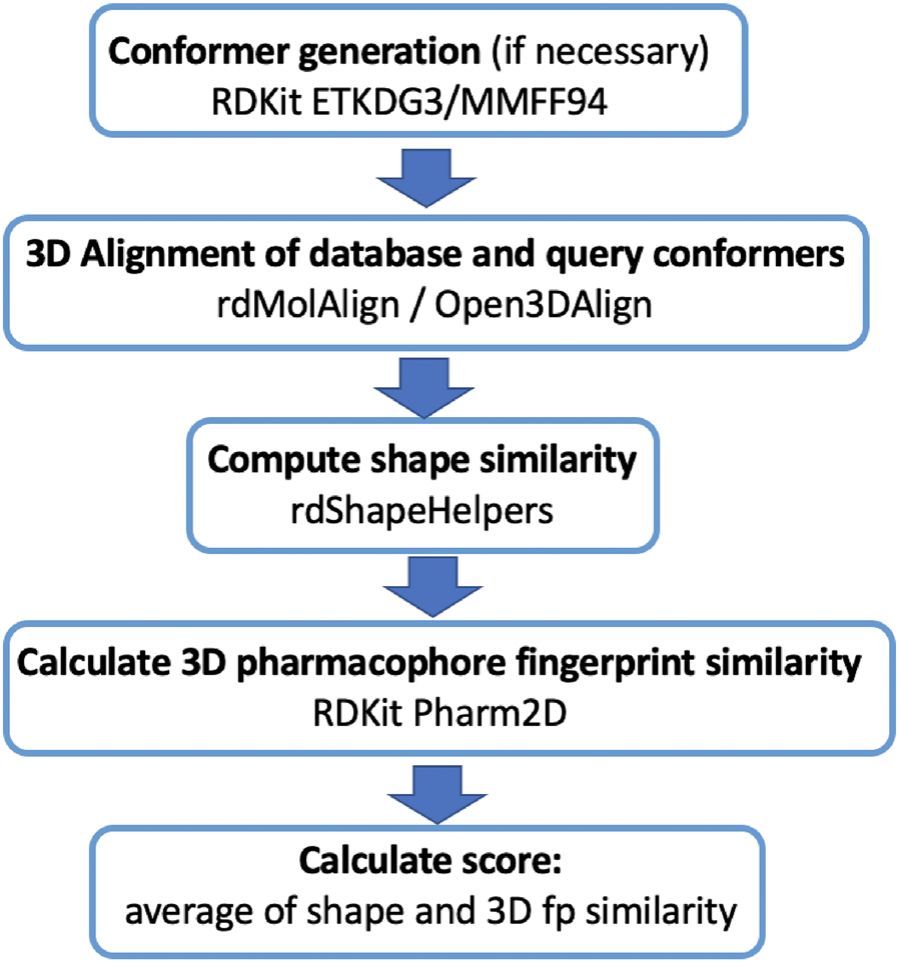
Different steps and RDKit functionalities which were combined to perform a screening based on pharmacophore alignment and shape similarity

Several functionalities of RDKit were combined to perform a screening based on a compounds’ molecular shape (Fig. 3). First, generation of conformers (RDKit ETKDGv3 and MMFF94 forcefield) is done for 2D query structures. Conformers for database compounds can be generated using the preparedb functionality. Then, conformers of each query molecule are aligned to all conformers of each database molecule with the RDKit Open3DAlign functionality, either using MMFF94 force field parameters or Crippen atomic logP contributions (user-defined). In the next step, for every conformer pair the shape similarity is calculated (TanimotoDist, TverskyShape or ProtrudeDist) and the most similar conformer pair for every query/database molecule pair is selected (RDKit rdShapeHelpers). For the selected most similar conformer pair a 3D pharmacophore fingerprint is generated (RDKit Pharm2D) and the fingerprint similarity is calculated. By default, a combined score (combo score), the average of shape similarity and 3D fingerprint similarity, is used to rank the database molecules. The intended use case of the shape screening mode is to screen a database of compounds with multiple conformers (prepared e.g. using the preparedb functionality of VSFlow) and to use a query ligand in a single, bioactive conformation, e.g. from the pdb database.

### managedb: manage databases

The mode managedb is a convenience tool to update and manage compound databases which are integrated into VSFlow. A detailed description can be found in the VSFlow wiki [26].

## Results and discussion

In the following section, the intended usage of VSFlow including some example commands are presented. A detailed description of the multiple possibilities to use VSFlow along with specific examples can be found in the VSFlow GitHub wiki [26].

In order to demonstrate the three main functionalities of VSFlow together with both its versatile input and output formats, we took the tyrosine-kinase inhibitor dasatinib as query molecule. As database, an SD file of the FDA-approved drugs generated from the ZINC database was used, comprising over 1600 molecules [14]. This database is also available in our GitHub repository.

### Substructure search

**Fig. 4:**
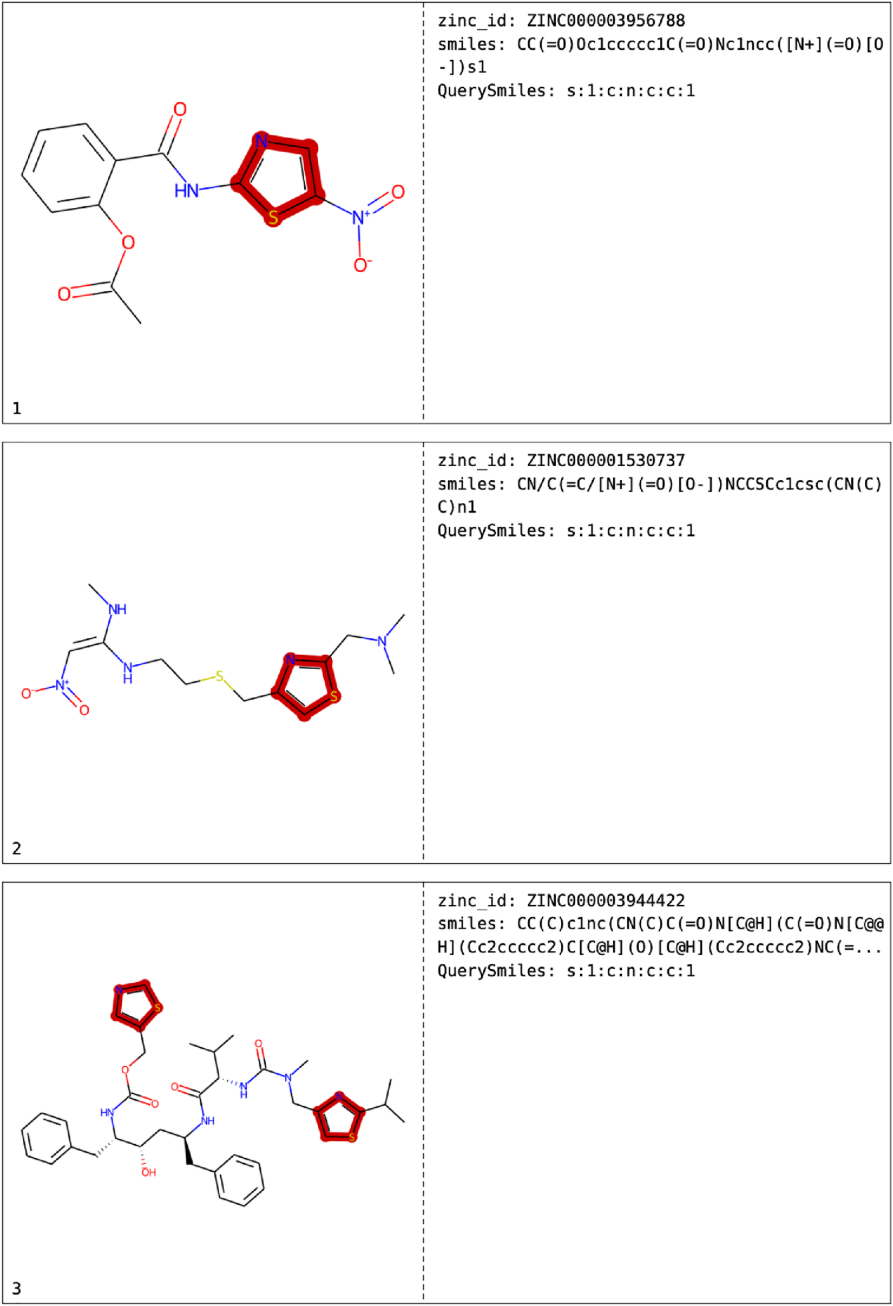
Examplary page of the pdf file generated after substructure search. The left column shows the hits with the substructure matches highlighted in red, the right column the ID of the hits as well as the SMILES and the query SMARTS

For the substructure search, a SMARTS representation of the thiazole function of dasatinib was taken as input to see how many other drugs might have that specific group. Besides the 36 hits (one of them, of course, dasatinib itself) in which the thiazole group was found, three molecules even have two thiazole groups, namely cefditoren, cobicistat and ritonavir. A pdf (supporting information) was generated displaying a table of the found hits with the 2D structures and the found substructure match highlighted in red as well as the information of the hit (e. g. ID, SMILES, Fig. 4). It should be mentioned that a pdf can only be generated in addition to an sdf, excel or csv file. 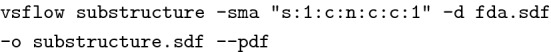

### Fingerprint similarity

**Fig. 5:**
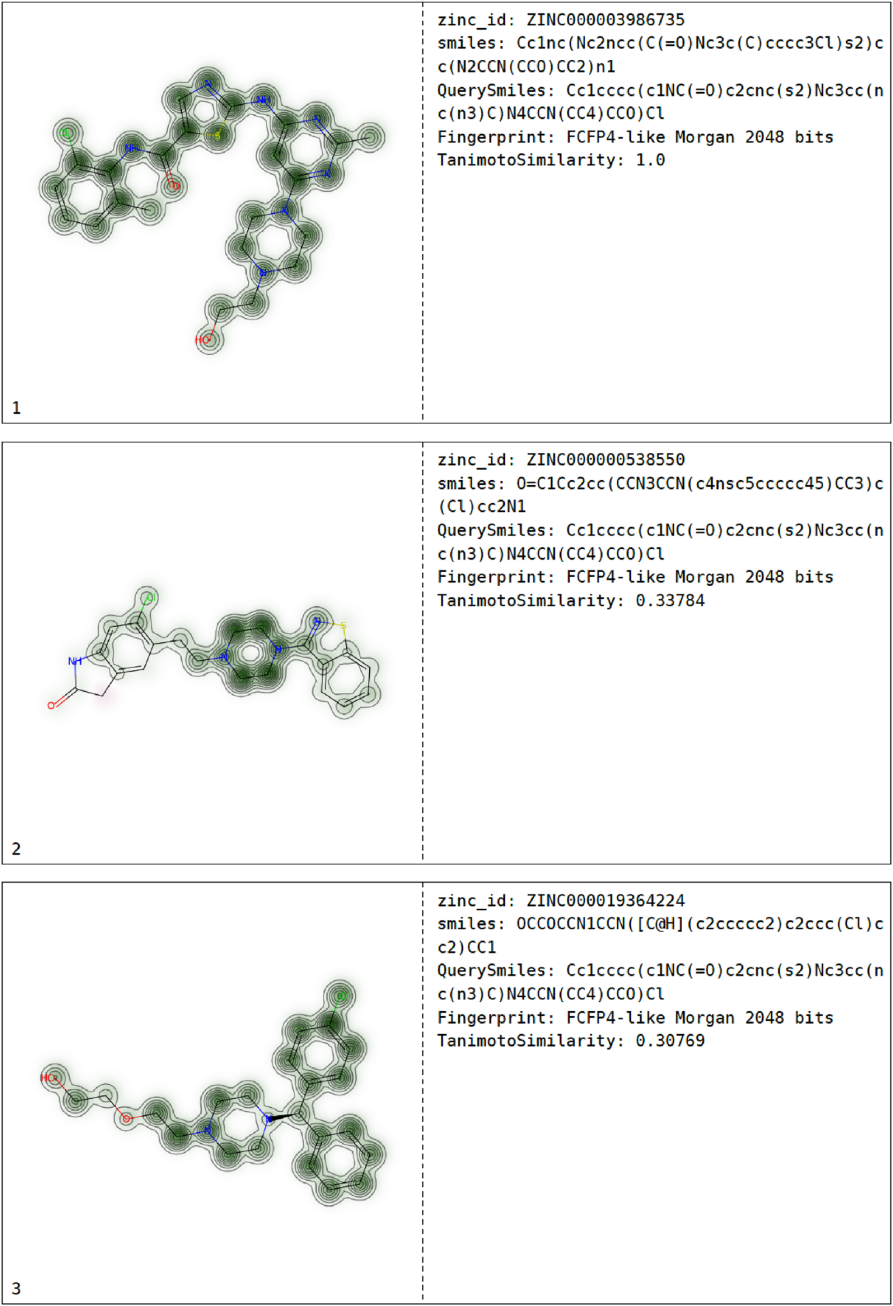
Examplary page of the pdf file generated after fpsim search. The fingerprint similarity (FCFP4-like Morgan 2048 bits) of the molecules with the query molecule dasatinib is visualized in the left column, the right column shows IDs of the molecule as well as the search parameters and the calculated Tanimoto similarity

For the fingerprint similarity function fpsim, a SMILES input of the molecule was used with default parameters, i. e. an FCFP4-like Morgan 2048 bits of radius 2 for which the Tanimoto coefficient was calculated. A pdf file was selected as output format as well as an Excel file. The simmap parameter will generate a similarity map that visualizes the contribution of the specific atoms to the similarity between the molecules in the database and dasatinib (Fig. 5) [27]. 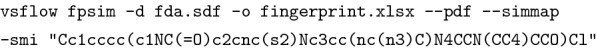

### Shape similarity

In order to perform a shape screening, a new database, containing a maximum of 20 conformers, was generated with the -c argument because the original database only had one conformer per compound. 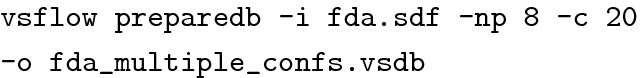


Since that is a rather resource-intensive step, multiprocessing was carried out with the help of the -np parameter. The following shape search, also multiprocessed, was then done with the previously prepared vsdb pickle file using the instance coordinates of dasatinib in complex with tyrosine protein kinase ABL1 (PDB: 2GQG). 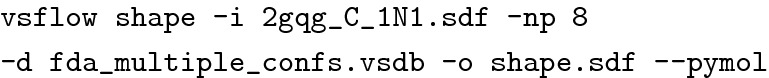


**Fig. 6:**
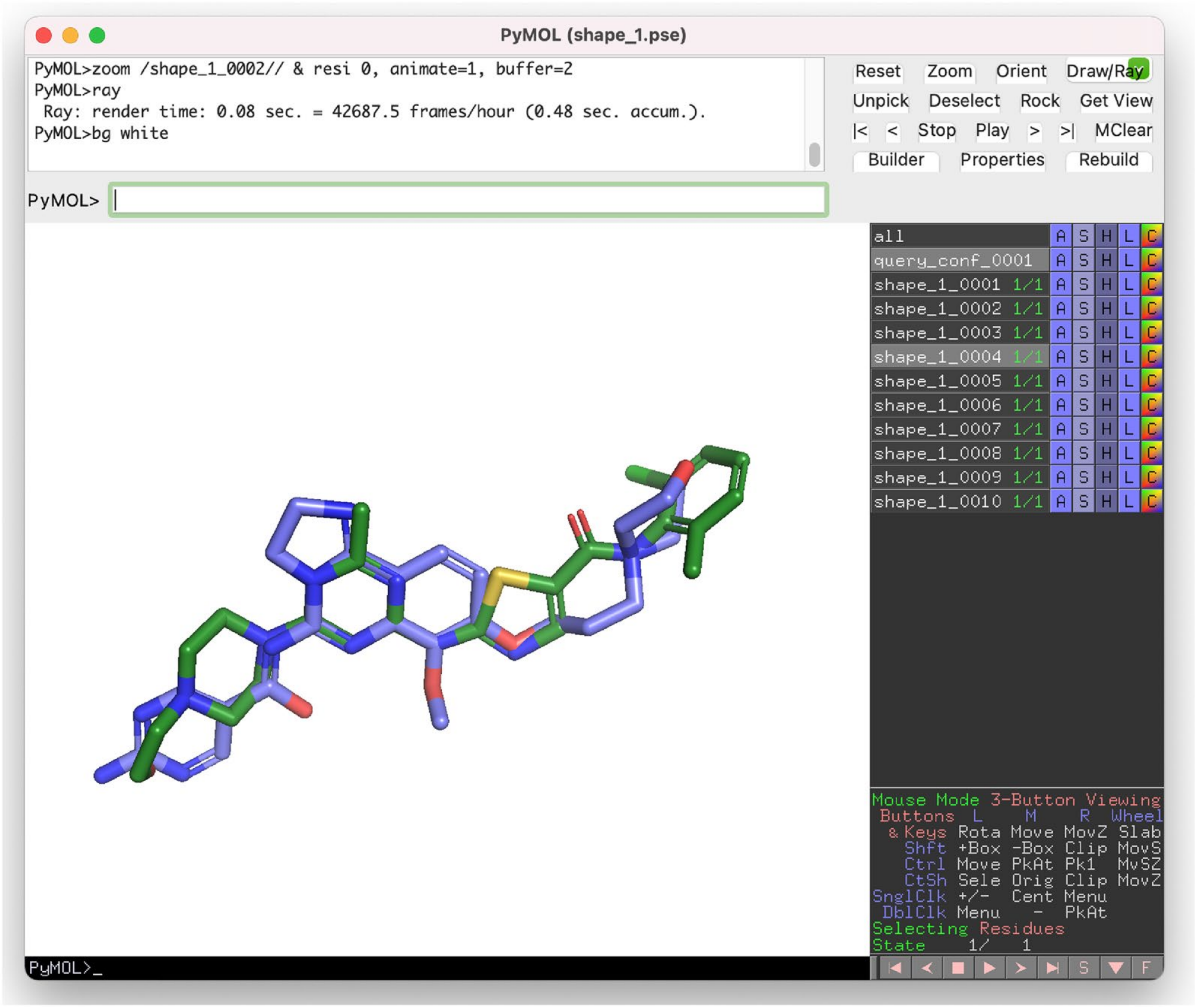
Screenshot from the PyMOL session file generated after shape similarity screening. By default, the first ten hits (one of them shown here in blue) are aligned with the query molecule dasatinib (green)

More than half of the top 10 hits were other kinase inhibitors. By default, the shape functionality creates two sd files, one with the query molecule (shape_1_query.sdf) and the found hits as a second file (shape_1.sdf). Additionally, a PyMOL session file was generated (--pymol parameter) so that the aligned structures could be visually inspected directly (Fig. 6).

**Fig. 7:**
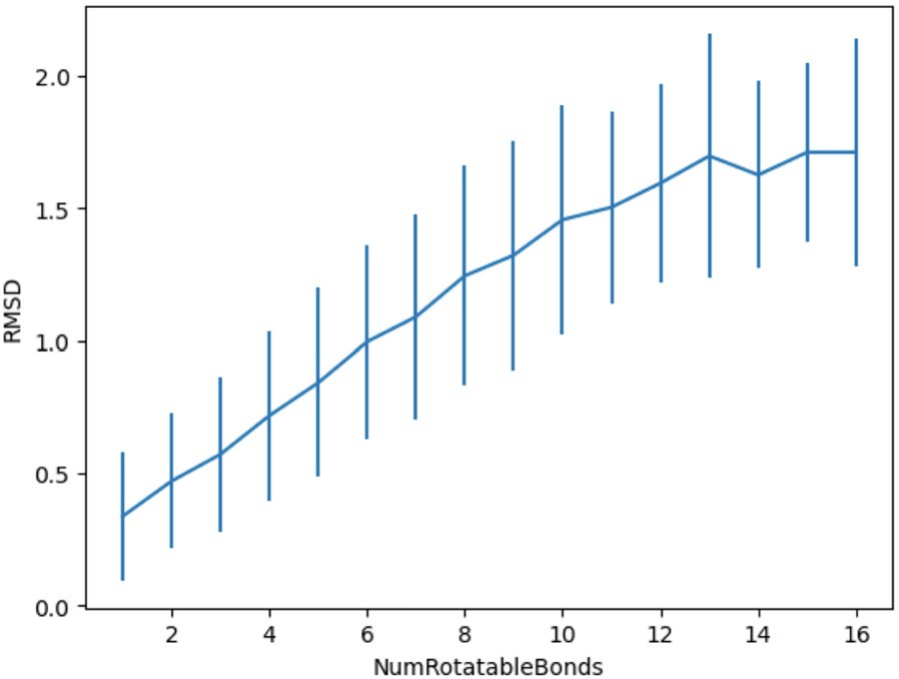
RMSD spread of the conformer generation process (ETKDG3 followed by MMFF94 minimization) for the search of the bioactive conformation (Platinum data set)

The RMSD spread of the conformer generation process (ETKDG3 followed by MMFF94 minimization) is given in Fig. 7). It shows a clear upwards trend: the more rotatable bonds, the larger the RMSD.

### Runtime performance

To give the user an idea of the expected runtime performance, we performed a substructure and 2D similarity search in the pdb and ChEMBL28 database [12, 28]. We performed the searches on up-to-date standard notebook hardware, namely a 12th Gen Intel(R) Core(TM) i7-12700 H with 2.70 GHz and 20 cores and 32GB RAM running Windows 11. To get an idea of the performance on your own system, you may execute the following commands accordingly. Both ChEMBL and pdb database can be downloaded and prepared directly within VSFlow: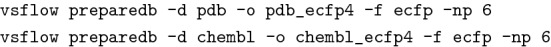 With the above calls, the pdb and chembl databases are downloaded into VSFlow and 2048-bit ECFP4 fingerprints are generated for each compound and stored within the output vsdb file. Preparation of the pdb database (containing 36,796 unique compounds at 22/05/2022) took 11 s on our system, preparation of the chembl28 database (2066377 compounds) took 511 s. Now, we performed a substructure and similarity screening using a SMILES as query, once in single-core mode and once on 6 cores: 
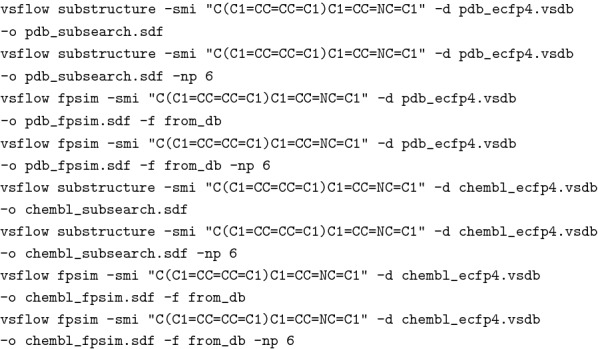
 Table 1 summarizes the overall runtime for each call, e.g. it contains the loading time for the database file, the substructure or similarity search and the generation of the output file.

**Table 1:** Runtime performance of substructure and similarity search on 12th Gen Intel(R) Core(TM) i7-12700 H with 2.70 GHz and 20 cores and 32GB RAM running Windows 11

	pdb database	ChEMBL database	cores
Substructure	1 s	162 s	1
	0.8 s	89 s	6
Similarity	1 s	157 s	1
	0.75 s	77 s	6

### Virtual screening performance

**Fig. 8:**
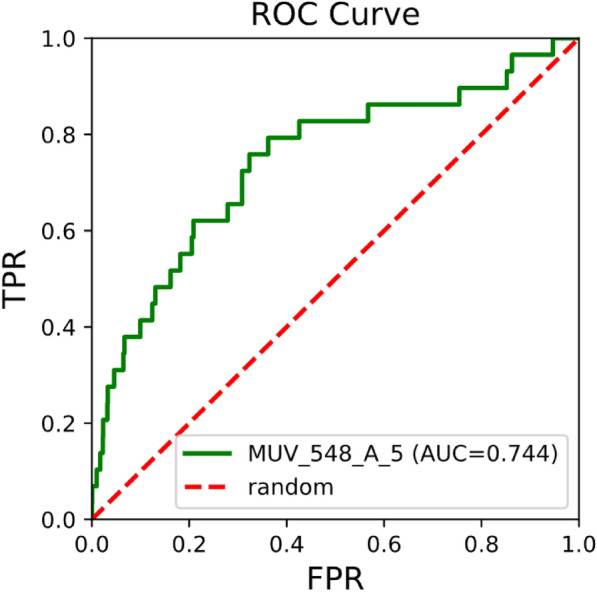
Example of a receiver operating curve (ROC) obtained for a query/validation test split after a 2D similarity screening with ECFP2 fingerprint. The MUV subset MUV_548 was the validation set, the query was compound MUV_548_A_5. The area under the curve (AUC) is 0.744. FPR = false positive rate, TPR = true positive rate

To give the user an idea about the performance of the tool in virtual screening practice, i.e. whether it could identify active compounds, we did some basic simulated screenings using the maximum unbiased validation (MUV) dataset [29]. The MUV dataset is based on PubChem bioactivity data and consists of 17 targets, each with 30 actives and 15,000 decoys. The choice of actives and decoys is done based on confirmatory and primary screens, which makes the dataset very difficult for virtual screening methods. We performed sample screenings based on 2D fingerprint and 3D shape similarity (mode fpsim and shape). The general performance of 2D fingerprints implemented in RDKit has been studied extensively before, with the MUV dataset being part of a larger evaluation set [30]. We adapted a simplified version of the workflow described before by Rohrer [29] and Riniker [30]. In short, for each of the 17 subsets in the MUV dataset, one of the 30 active compounds was selected as query molecule and the remaining 29 actives were pooled together with the 15,000 decoys and used as validation set. This query/validation split was done for all 30 actives. For the resulting 30 query/validation test splits per subset the virtual screening performance was measured by the area under the receiver operating curve (AUC, example curve shown in Fig. 8) and the mean value was calculated for each subset (mean AUC). The screening consisted of two steps: (1) generation of a vsdb database with standardized molecules and pre-computed fingerprints or conformers for the validation set; (2) 2D or 3D similarity screening of the validation set against the query molecule.

**Fig. 9:**
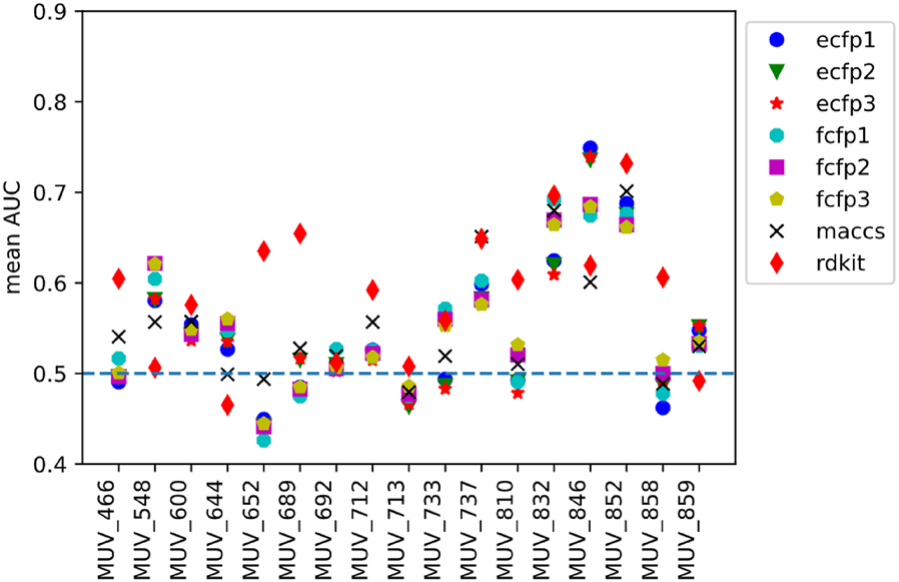
Results of virtual screening validation with the MUV dataset for 2D fingerprint similarity. The expectation of mean AUC of 0.5 for random rankings is indicated by the blue dashed line

The results for 2D similarity screening with various descriptors is summarized in Fig. 9. They follow, in general, the trend observed by Riniker et al. for 2D fingerprints on the MUV dataset [30]. For some targets, a significant enrichment of actives (e.g. meanAUC = 0.74 for ECFP3 fingerprint for target FactorXIa [MUV_846]) is observed, whereas for other targets no enrichments could be observed based on simple 2D similarty calculations.

**Fig. 10:**
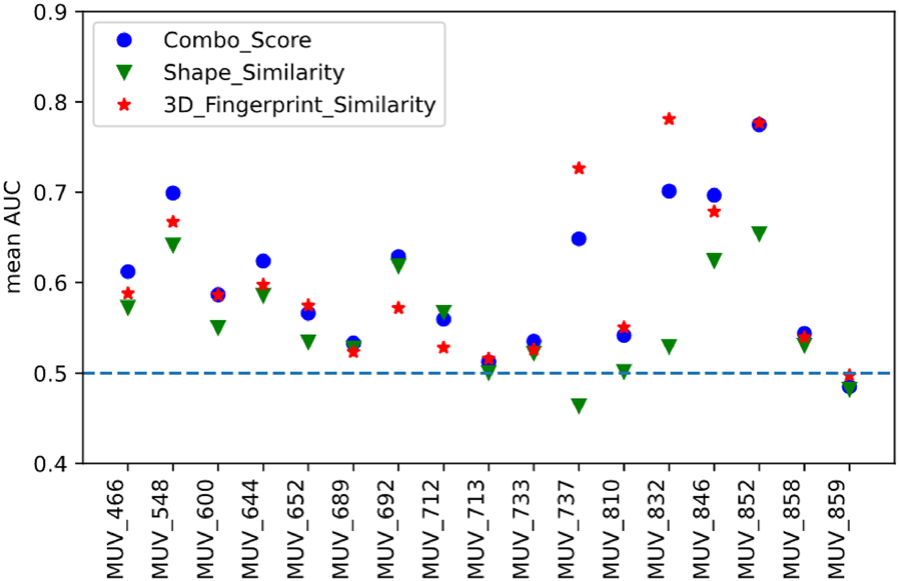
Results of virtual screening validation with the MUV dataset for 3D shape-based screenings. The expectation of mean AUC of 0.5 for random rankings is indicated by the blue dashed line

Fig. 10 summarizes the results for the 3D shape-based virtual screenings. Best performance is observed when using the combo score for result ranking for most MUV subsets. However, for MUV_737 (estrogen receptor alpha) and MUV_832 (cathepsin G) scoring with 3D fingerprint yields a better overall enrichment.

## Conclusions

VSFlow is a versatile command-line tool to perform ligand-based virtual screenings in large compound databases on the basis of the RDKit cheminformatics framework. It allows to perform a substructure search, a 2D fingerprint-based and a 3D shape-based similarity search based on the respective functionalities implemented in RDKit. Screenings can be easily parallelized to multiple cores and the screening results can be directly visualized as pdf or pymol file. The integration of VSFlow in existing virtual screening setups is straightforward because the entire code is open source.

## Availability and requirements

- Project name: VSFlow - Virtual Screening Workflow
- Project home page: https://github.com/czodrowskilab/VSFlow
- Operating system(s): Platform independent
- Programming language: Python
- Other requirements: Anaconda or Miniconda
- License: MIT
- Any restrictions to use by non-academics: no.

## Data Availability

The source code and the files needed to reproduce the examples from this manuscript can be found at https://github.com/czodrowskilab/VSFlow.

## References

[1] Maia EHB, Assis LC, de Oliveira TA, da Silva AM, Taranto AG (2020). Structure-based virtual screening: from classical to artificial intelligence. Front Chem.

[2] Gimeno A, Ojeda-Montes MJ, Tomás-Hernández S, Cereto-Massagué A, Beltrán-Debón R, Mulero M, Pujadas G, Garcia-Vallvé S (2019). The light and dark sides of virtual screening: what is there to know?. Int J Mol Sci.

[3] Maggiora G, Vogt M, Stumpfe D, Bajorath J (2014). Molecular similarity in medicinal chemistry. J Med Chem.

[4] ROCS 3.4.3.0: OpenEye Scientific Software, Santa Fe, NM (2022) http://www.eyesopen.com. Accessed 7 Apr 2022

[5] Torres PHM, Sodero ACR, Jofily P, Silva-Jr FP (2019). Key topics in molecular docking for drug design. Int J Mol Sci.

[6] Pinzi L, Rastelli G (2019). Molecular docking: shifting paradigms in drug discovery. Int J Mol Sci.

[7] Gentile F, Yaacoub JC, Gleave J, Fernandez M, Ton AT, Ban F, Stern A, Cherkasov A (2022). Artificial intelligence-enabled virtual screening of ultra-large chemical libraries with deep docking. Nat Protoc.

[8] Warr WA, Nicklaus MC, Nicolaou CA, Rarey M (2022). Exploration of ultralarge compound collections for drug discovery. J Chem Inf Model.

[9] Lessel U, Wellenzohn B, Lilienthal M, Claussen H (2009). Searching fragment spaces with feature trees. J Chem Inf Model.

[10] infiniSee version 4.0.0; BioSolveIT GmbH, Sankt Augustin, Germany (2022) www.biosolveit.de/infiniSee

[11] Singh N, Chaput L, Villoutreix BO (2021). Virtual screening web servers: designing chemical probes and drug candidates in the cyberspace. Brief Bioinform.

[12] Gaulton A, Hersey A, Nowotka ML, Bento AP, Chambers J, Mendez D, Mutowo P, Atkinson F, Bellis LJ, Cibrian-Uhalte E, Davies M, Dedman N, Karlsson A, Magarinos MP, Overington JP, Papadatos G, Smit I, Leach AR (2017). The chembl database in 2017. Nucleic Acids Res.

[13] Kim S, Chen J, Cheng T, Gindulyte A, He J, He S, Li Q, Shoemaker BA, Thiessen PA, Yu B, Zaslavsky L, Zhang J, Bolton EE (2021). Pubchem in 2021: new data content and improved web interfaces. Nucleic Acids Res.

[14] Sterling T, Irwin JJ (2015). Zinc 15 - ligand discovery for everyone. J Chem Inf Model.

[15] Bragina ME, Daina A, Perez MAS, Michielin O, Zoete V (2022). The swisssimilarity 2021 web tool: Novel chemical libraries and additional methods for an enhanced ligand-based virtual screening experience. Int J Mol Sci.

[16] Zoete V, Daina A, Bovigny C, Michielin O (2016). Swisssimilarity: a web tool for low to ultra high throughput ligand-based virtual screening. J Chem Inf Model.

[17] Sunseri J, Koes DR (2016). Pharmit: interactive exploration of chemical space. Nucleic Acids Res.

[18] Dalke A (2019). The chemfp project. J Cheminform.

[19] https://github.com/schrodinger/gpusimilarity. Accessed 22 May 2022

[20] https://www.nextmovesoftware.com/arthor.html. Accessed 22 May 2022

[21] RDKit: Open-Source Cheminformatics Software (2022). https://www.rdkit.org. Accessed 7 Apr 2022

[22] Anaconda Software Distribution. Anaconda Inc (2020). https://docs.anaconda.com/

[23] https://github.com/mcs07/MolVS.Accessed 22 May 2022

[24] The PyMOL Molecular Graphics System, Version 2.0 Schrödinger, LLC (2022). https://pymol.org

[25] Wang S, Witek J, Landrum GA, Riniker S (2020). Improving conformer generation for small rings and macrocycles based on distance geometry and experimental torsional-angle preferences. J Che Inf Model.

[26] https://github.com/czodrowskilab/VSFlow/wiki. Accessed 22 May 2022

[27] Riniker S, Landrum GA (2013). Similarity maps—a visualization strategy for molecular fingerprints and machine-learning methods. J Cheminform.

[28] Berman HM, Westbrook J, Feng Z, Gilliland G, Bhat TN, Weissig H, Shindyalov IN, Bourne PE (2000). The protein data bank. Nucleic Acids Res.

[29] Rohrer SG, Baumann K (2009). Maximum unbiased validation (muv) data sets for virtual screening based on pubchem bioactivity data. J Chem Inf Model.

[30] Riniker S, Landrum GA Open-source Platform to Benchmark Fingerprints for Ligand-based Virtual Screening. http://www.jcheminf.com/content/5/1/26 10.1186/1758-2946-5-26 PMC3686626 23721588

